# Addison's disease presenting with idiopathic intracranial hypertension in 24-year-old woman: a case report

**DOI:** 10.1186/1752-1947-4-60

**Published:** 2010-02-19

**Authors:** Dushyant Sharma, Rohini Mukherjee, Peter Moore, Daniel J Cuthbertson

**Affiliations:** 1 Department of Diabetes and Endocrinology, Clinical Sciences Centre, University Hospital Aintree, Liverpool L9 7AL, UK; 2 Department of Neurology, Walton Centre for Neurology and Neurosurgery NHS Trust, Liverpool L9 7AL, UK

## Abstract

**Introduction:**

Idiopathic intracranial hypertension can rarely be associated with an underlying endocrine disorder such as Cushing's syndrome, hyperthyroidism, or with administration of thyroxine or growth hormone. Though cases of idiopathic intracranial hypertension associated with Addison's disease in children have been reported, there is only one documented case report of this association in adults. We describe a case of an acute adrenal insufficiency precipitated by idiopathic intracranial hypertension in a Caucasian female.

**Case presentation:**

A 24-year-old Caucasian woman was acutely unwell with a background of several months of generalised fatigue and intermittent headaches. She had unremarkable neurological and systemic examination with a normal computerised tomography and magnetic resonance imaging of the brain. Normal cerebrospinal fluid but increased opening pressure at lumbar puncture suggested intracranial hypertension. A flat short synacthen test and raised level of adrenocorticotrophic hormone were consistent with primary adrenal failure.

**Conclusion:**

Addison's disease can remain unrecognised until precipitated by acute stress. This case suggests that idiopathic intracranial hypertension can rarely be associated with Addison's disease and present as an acute illness. Idiopathic intracranial hypertension is possibly related to an increase in the levels of arginine vasopressin peptide in serum and cerebrospinal fluid secondary to a glucocorticoid deficient state.

## Introduction

Idiopathic intracranial hypertension (IIH) describes the clinical syndrome of raised intracranial pressure, in the absence of space-occupying lesions or vascular lesions, without enlargement of the cerebral ventricles, for which no causative factor can be identified [1]. The condition is frequently associated with obesity or with various drugs including antibiotics (tetracyclines, nitrofurantoin, nalidixic acid), amiodarone, cyclosporin, systemic and topical steroids or the oral contraceptive pill. However, IIH is rarely associated with underlying endocrine disorders such as Cushing's syndrome, hyperthyroidism or with the administration of thyroxine or growth hormone. We describe the case of a woman presenting with acute chronic adrenal insufficiency associated with IIH. The pathophysiological mechanism proposed is that the gluco- and mineralocorticoid deficient state is accompanied by a sustained overproduction of anti-diuretic hormone (ADH) causing intracranial hypertension.

## Case presentation

A 24-year-old Caucasian woman was admitted to the Accident and Emergency Department of our hospital with a sudden episode of nausea, vomiting and collapse, having become acutely unwell whilst at work. There was no past medical history and she was not taking any regular medication. On examination she was of normal body weight (weight 53 kg and body mass index 21 kg/m^2^), afebrile and her blood pressure was 103/56 with no postural change measured. Although she appeared drowsy and unwell, her systemic examination was unremarkable and no focal abnormalities were found on neurological examination of the central or peripheral nervous system. Initial biochemical analysis revealed sodium 127 mmol/l, potassium 3.2 mmol/l, urea 3.8 mmol/l, creatinine 77 and glucose 4.1 mmol/l. Inflammatory markers (white cell count and C-reactive protein) were normal. A computerised tomography (CT) and magnetic resonance (MR) of the brain demonstrated normal ventricles, no focal lesion or mass effect and normal sagittal sinus flow. Upon lumbar puncture, performed in the lateral decubitus position, an opening pressure of 40 mm of water was documented. Cerebrospinal fluid (CSF) microscopy revealed two white blood cells (WBCs) per mm^3^, <1 red blood cell (RBC) per mm^3 ^and no organisms. CSF chemistry was unremarkable: protein 0.4 g/l and glucose 3.2 mmol/l. The patient had remained drowsy and she was managed in the Intensive Care Unit with a presumptive diagnosis of acute meningoencephalitis with a secondary syndrome of inappropriate anti-diuretic hormone (SIADH). No measurements of serum or urine osmolality were made. She was treated with antibiotics and acyclovir and was also supported with intravenous fluids and, within 24 hours, had improved such that she was transferred back to the ward.

**Figure 1:**
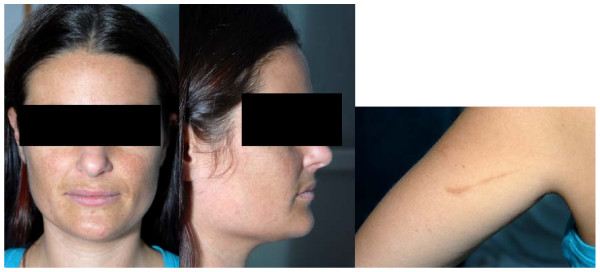
**Facial and scar pigmentation**.

During subsequent review she admitted to several months of generalised fatigue, sometimes falling asleep at work, and of intermittent headaches. With subsequent neurology specialist input to review the grossly elevated intracranial pressure, accompanied by normal imaging and CSF analysis, the patient was commenced on acetazolamide 250 mg daily for presumed idiopathic intracranial hypertension (IIH). Visual acuity and visual field (Goldman perimetry) testing was unremarkable. On endocrine review, generalised hyperpigmentation with facial melasma were noted and, although she had no buccal pigmentation, she did have marked pigmentation of a recent scar over her left shoulder (Figure 1). A short synacthen (250 mcg) test (SST) was performed and demonstrated adrenal insufficiency with basal cortisol at 231 nmol/l, 30 minute cortisol 265 nmol/l and 60 minute at 200 nmol/l. A repeat SST showed basal cortisol at 138 nmol/l and 30 minute cortisol at 159 nmol/l and confirmed adrenal insufficiency. Adrenal antibodies were negative. Her plasma adrenocorticotropic hormone (ACTH) was raised at >278 pmol/l consistent with primary adrenal failure. She was commenced on glucocorticoid (hydrocortisone 10 mg bd) and mineralocorticoid (fludrocortisone 50 mcg) replacement therapy and discharged. On subsequent review two weeks later, she was feeling much better with CSF pressure reduced to 25 mm of water on repeat lumbar puncture. Acetazolamide was discontinued after three months and on subsequent reviews at six and 12 months, she continued to remain well on hydrocortisone and fludrocortisone replacement.

## Discussion

Idiopathic intracranial hypertension is defined as the clinical syndrome of raised intracranial pressure, in the absence of space-occupying lesions or vascular lesions, without enlargement of the cerebral ventricles, for which no causative factor can be identified [1]. Although IIH is often associated with papilloedema, papilloedema is not an absolute requirement to make the diagnosis. Historically IIH was referred to as pseudotumour cerebri as it mimics an intracranial tumour. More recently, it has been referred to as benign intracranial hypertension although this term has also been abandoned because a small but significant number of patients develop visual impairment or visual loss. However, even the current term idiopathic intracranial hypertension is inaccurate with the condition frequently associated with obesity or with the use of medication including various antibiotics (tetracyclines, nitrofurantoin, and nalidixic acid), amiodarone, cyclosporin, systemic and topical steroids, and the oral contraceptive pill. Of relevance, various endocrine disorders have also rarely been reported in association with otherwise idiopathic intracranial hypertension including Cushing's syndrome [2], hyperthyroidism [3] as well as the administration of thyroxine or growth hormones [4]. There has only been one previous documented case of idiopathic intracranial hypertension occurring in association with Addison's disease in an adult [5] with two further cases reported in children [6].

Although the pathophysiology of IIH is uncertain, the mechanisms that have been proposed for its development include increased production of CSF, reduced CSF absorption, or increased cerebral venous pressure causing a secondary increase in CSF pressure. Analysis of CSF arginine vasopressin (AVP) in patients with IIH demonstrates it to be elevated compared to healthy controls [7]. This would seem to correlate with reports that patients with glucocorticoid deficiency have increased plasma levels of AVP and a sustained hypersecretion of AVP despite plasma dilution [8]. Thus it is possible that increased serum, and possibly CSF AVP may mediate IIH in Addison's disease.

In this case, there are two weaknesses to acknowledge with regards to demonstrating the likely association between Addison's disease and intracranial hypertension. Firstly, we were unable to measure serum or CSF AVP to provide the mechanistic link. Secondly, the patient's intracranial hypertension was treated with acetazolamide and did not necessarily reduce solely as a consequence of steroid replacement. However, standard treatment for IIH was instituted in addition to steroid replacement to minimise any risk of visual loss.

## Conclusion

Addison's disease can remain unrecognised for a long time until acute adrenal insufficiency is precipitated by an acute stress. This case suggests that IIH can rarely be associated with Addison's disease and presents as an acute illness. The association of IIH with Addison's disease is possibly secondary to increased serum and CSF arginine vasopressin peptide (AVP) in a glucocorticoid deficient state. Though standard treatment of IIH is acetazolamide, replacing steroids on identifying Addison's disease as the cause for the condition might reduce the risk of loss of vision and provide early symptom relief.

## Abbreviations

IIH: idiopathic intracranial hypertension; ADH: anti-diuretic hormone; CT: computerised tomography; MR: magnetic resonance; CSF: cerebrospinal fluid; WBC: white blood cells; RBC: red blood cells; SIADH: syndrome of inappropriate anti-diuretic hormone; ACTH: adreno-corticotrophic hormone; AVP: arginine vasopressin.

## Consent

Written informed consent was obtained from the patient for the publication of this case report and accompanying images. A copy of the written consent is available for review by the Editor-in-chief of the journal.

## Competing interests

The authors declare that they have no competing interests.

## Authors' contributions

RM organised the various investigations, collected information and conducted the literature search. PM provided the neurological evaluation while DJC and DS provided the endocrine evaluation along with a contribution in writing the manuscript. All authors read and approved the final manuscript.
