# Correction: Addison's disease presenting with idiopathic intracranial hypertension in 24-year-old woman: a case report

**DOI:** 10.1186/1752-1947-4-362

**Published:** 2010-11-15

**Authors:** Dushyant Sharma, Rohini Mukherjee, Peter Moore, Daniel J Cuthbertson

**Affiliations:** 1 Department of Diabetes and Endocrinology, Clinical Sciences Centre, University Hospital Aintree, Liverpool L9 7AL, UK; 2 Department of Neurology, Walton Centre for Neurology and Neurosurgery NHS Trust, Liverpool L9 7AL, UK

## Correction

Following the publication of our article [1] an error in the case presentation section was noted. In the description of the lumbar puncture procedure, we incorrectly stated the units of the opening pressure.

Upon lumbar puncture, performed in the lateral decubitus position, an opening pressure of 40 mm of water was documented.

Should read:

Upon lumbar puncture, performed in the lateral decubitus position, an opening pressure of 40 cm of water was documented.

## References

[B1] Sharma D Mukherjee R Moore P Cuthbertson DJ Addison's disease presenting with idiopathic intracranial hypertension in 24-year-old woman: a case report Journal of Medical Case Reports 2010 4 60 10.1186/1752-1947-4-60 20170523 PMC2848063

